# Meyer's loop asymmetry and language lateralisation in epilepsy

**DOI:** 10.1136/jnnp-2015-311161

**Published:** 2015-09-18

**Authors:** Mark Nowell, Sjoerd B Vos, Meneka Sidhu, Kaitlin Wilcoxen, Narek Sargsyan, Sebastien Ourselin, John S Duncan

**Affiliations:** 1 Department of Clinical and Experimental Epilepsy, UCL Institute of Neurology, London, UK; 2 MRI Unit, Epilepsy Society, Chalfont St Peter, UK; 3 Translational Imaging Group, Centre of Medical Imaging and Computing, UCL, London, UK

## Abstract

**Objectives:**

Several studies have suggested an asymmetry in Meyer's loop in individuals, with the left loop anterior to the right. In this study we test the hypothesis that there is an association between Meyer's loop asymmetry (MLA) and language lateralisation.

**Methods:**

57 patients with epilepsy were identified with language functional MRI (fMRI) and diffusion MRI acquisition. Language lateralisation indices from fMRI(LI) and optic radiation and arcuate fasciculus probabilistic tractography was performed for each subject. The subjects were divided into left language dominant (LI>0.4) and non-left language groups (LI<0.4) according to their LI.

**Results:**

A negative linear correlation was identified between language lateralisation and MLA, with greater left lateralised language associated with more anteriorly placed left Meyer's loops (R value −0.34, p=0.01). There was a significant difference in mean MLA between the two groups, with the left loop being anterior to the right loop in the LI>0.4 group and posterior to the right loop in the LI<0.4 group (p=0.003). No correlation was found between language lateralisation and arcuate fasciculus volume.

**Conclusions:**

This study suggests an association between the extent of Meyer's loop asymmetry and the lateralisation of language determined by fMRI in patients with epilepsy. Further studies should be carried out to evaluate this association in control subjects and with other measures of language lateralisation.

## Introduction

White matter connexions can be delineated and quantified in vivo using diffusion MRI and tractography.^1^ ^2^ Asymmetry of white matter tracks is well-described, with larger left than right corticospinal tracts in group analyses.^3^

The literature on arcuate fasciculus asymmetry is mixed. In individual subjects, some studies have shown a correlation between the volume of the arcuate fasciculus tract and language lateralisation, determined by the WADA test^4^ and by functional MRI (fMRI)I,^5^ while others have not found a consistent association.^6^ Recent work suggests the fasciculus may in fact be composed of two parallel tracts, with independent associations to language dominance.^3^ ^7^

Meyer's loop is the anterior bundle of the optic radiation, a white matter tract that makes up part of the visual pathway. Meyer's loop runs from the lateral geniculate nucleus of the thalamus, passes anteroinferiorly over the temporal horn of the lateral ventricle and then sharply turns posteriorly to join the central and posterior bundles of the optic radiation heading towards the occipital cortex. There is considerable interindividual variability in the anterior extent of Meyer's loop,^8^ as shown in both anatomical studies (22–37 mm from the temporal pole^9^) and probabilistic tractography (24–47 mm from the temporal pole^10^). Meyer's loop is at risk with anterior temporal lobe resections, and is therefore of great interest in epilepsy surgery.

In a recent study deterministic tractography was applied to 20 healthy right-handed volunteers, to look for asymmetry in the anterior extent of Meyer's loop in individual subjects.^11^ A significant asymmetry was found, with mean Meyer's loop-temporal pole distances measured as 39.7 mm on the left and 45.5 mm on the right. This asymmetry is supported by probabilistic tractography studies, which have similarly reported a trend towards more anteriorly placed Meyer's loops in the left temporal lobe.^10^ This concurs with the finding that visual field defects are 3.5 times more likely with left-sided than right-sided anterior temporal lobe resections.^12^

In this study we test the hypotheses that: 1. Asymmetries in the anterior extent of Meyer's loop are associated with language lateralisation, as the structural correlates associated with language development may result in displacement of Meyer's loop to a more anterior location. 2. Arcuate fasciculus volume is correlated with language lateralisation in individuals with focal epilepsy.

## Methods

### Subjects and recruitment

This project was approved by the Joint Research Ethics Committee of the National Hospital for Neurology and Neurosurgery (NHNN), and University College London (UCL) Institute of Neurology (ION). All participants were provided with patient information sheets and gave written, informed consent.

All subjects had medically refractory focal epilepsy, and were undergoing presurgical evaluation at the National Hospital for Neurology and Neurosurgery. Subjects were selected based on the acquisition of language fMRI studies and diffusion MRI acquisition at the National Epilepsy Society (ES) between August 2010 and August 2013. The group was selected to give an enriched, roughly even distribution of left and non-left language dominance. This enables a testing of the hypothesis, but is not reflective of the normal population, in whom language is predominantly on the left side.

Sixty-one patients were identified with language fMRI and diffusion MRI acquisition. Four patients were excluded from further analysis as they had large mass lesions that distorted the location of Meyer's loop. The demographics of the remaining 57 patients are shown in table 1. Fifty-four patients had a diagnosis of temporal lobe epilepsy, and three patients had extratemporal epilepsy. The majority of subjects did have radiological lesions shown on imaging, but none had structural pathology that affected the location of Meyer's loop. For the purposes of this study, left language dominance is defined by fMRI as a lateralisation index of 0.4 or above using the verbal fluency paradigm and non-left language dominance is defined as a lateralisation index of below 0.4.^13^ ^14^

**Table 1:** Demographics of study population

Language	Number	Median age (range)	Sex (M/F)	Median duration of epilepsy (range) years	Side of epilepsy (R/L)	Lesional	Handed (R/L)
Non-left	21	38 (20–56)	10/11	16 (1–33)	11/10	TOTAL—18 (86%) DNET—5 HS—11 FCD—2	11/10
Left	36	38 (19–58)	14/22	17 (1–44)	14/22	TOTAL—30 (83%) DNET—6 HS—19 FCD—2 Dual path—2 Cavernoma—1	31/5

DNET, dysembryoplastic neuroepithelial tumour; Dual path, dual pathology; FCD, focal cortical dysplasia; F, female; HS, hippocampal sclerosis; L, left; M, male; R, right;

### MRI acquisition

fMRI and diffusion MRI were performed on a 3 T GE Excite II scanner (General Electric, Waukesha, Milwaukee, Wisconsin, USA).

For fMRI, gradient echo echoplanar images provided blood oxygen level-dependent contrast, and each volume comprised 36 contiguous oblique axial slices of 2.4 mm thickness. The field of view of 24×24 cm was acquired with a 64×64 matrix that was zero-filled to 64×64 for a voxel size of 3.75×3.75×2.5 mm. Sequence timings were TE/TR=22./2500 ms, with parallel imaging factor 2.

For diffusion MRI, data were acquired using a cardiac-triggered single-shot spin-echo echo planar imaging sequence with echo time (TE) of 74.7 ms, with parallel imaging factor 2. Sixty contiguous axial slices of 2.4 mm thickness were obtained covering the whole brain, and diffusion-weighting gradients were applied in 52 non-collinear directions^15^ with a b-value of 1200 s/mm^2^, along with six non-diffusion-weighted scans. The field of view of 24×24 cm was acquired with a 96×96 matrix that was zero-filled to 128×128 for a voxel size of 1.875×1.875×2.4 mm.

In addition, a three-dimensional (3D) T1-weighted Fast Spoiled Gradient-Recalled (FSPGR) image was acquired with a field of view of 187×240×240 mm (AP×LR×IS) and a 170×256×256 acquisition matrix for a 1.1×0.94×0.94 mm voxel size. Sequence details include: TE/TR/TI=3.06/8.14/450 ms, flip angle 20°, parallel imaging acceleration factor 2.

### fMRI language paradigm

Each subject performed a verbal fluency language task. This consists of a blocked experimental design with 30 s activation blocks alternating with 30 s of cross-hair fixation during the baseline condition over 5.5 min. This paradigm is known to reliably lateralise expressive language.^16^ For this paradigm subjects were asked during the activation phase to covertly generate different words beginning with a visually presented letter (A, S, W, D and E) contrasted by crosshair fixation as rest condition. This paradigm was used to identify language regions in the inferior and middle frontal gyri.^17^ ^18^

### fMRI preprocessing and LI calculation

The verbal fluency paradigm, used in clinical practice,^19^ was analysed using SPM8 (http://www.fil.ion.ucl.ac.uk/spm/). Imaging time series were realigned, normalised into standard anatomical space and smoothed. An anatomical mask incorporating the inferior frontal and middle frontal gyri was used. A bootstrap method was employed to calculate lateralisation indices (LI) within the mask in all patients using the SPM8 LI toolbox.

### Diffusion preprocessing

The diffusion MRI were transferred to a Linux workstation, and corrected for eddy current correction distortion using the eddy _correct tool in FSL.^20^ Data was processed using the MRtrix software package 0.2.12 (http://www.brain.org.au/software/) to produce a fractional anisotropy (FA) map, directionally-encoded colour map and constrained spherical deconvolution (CSD) fibre orientation distributions.^21–24^ CSD is a higher order model for estimating fibre orientations, which uses high angular resolution diffusion-weighted imaging data and can resolve multiple fibre populations within each imaging voxel. We used a maximum harmonic order (Lmax) of 8, and obtained single fibre response functions from all voxels in the brain with FA values of 0.7 or higher.

### Tractography

**Figure 1:**
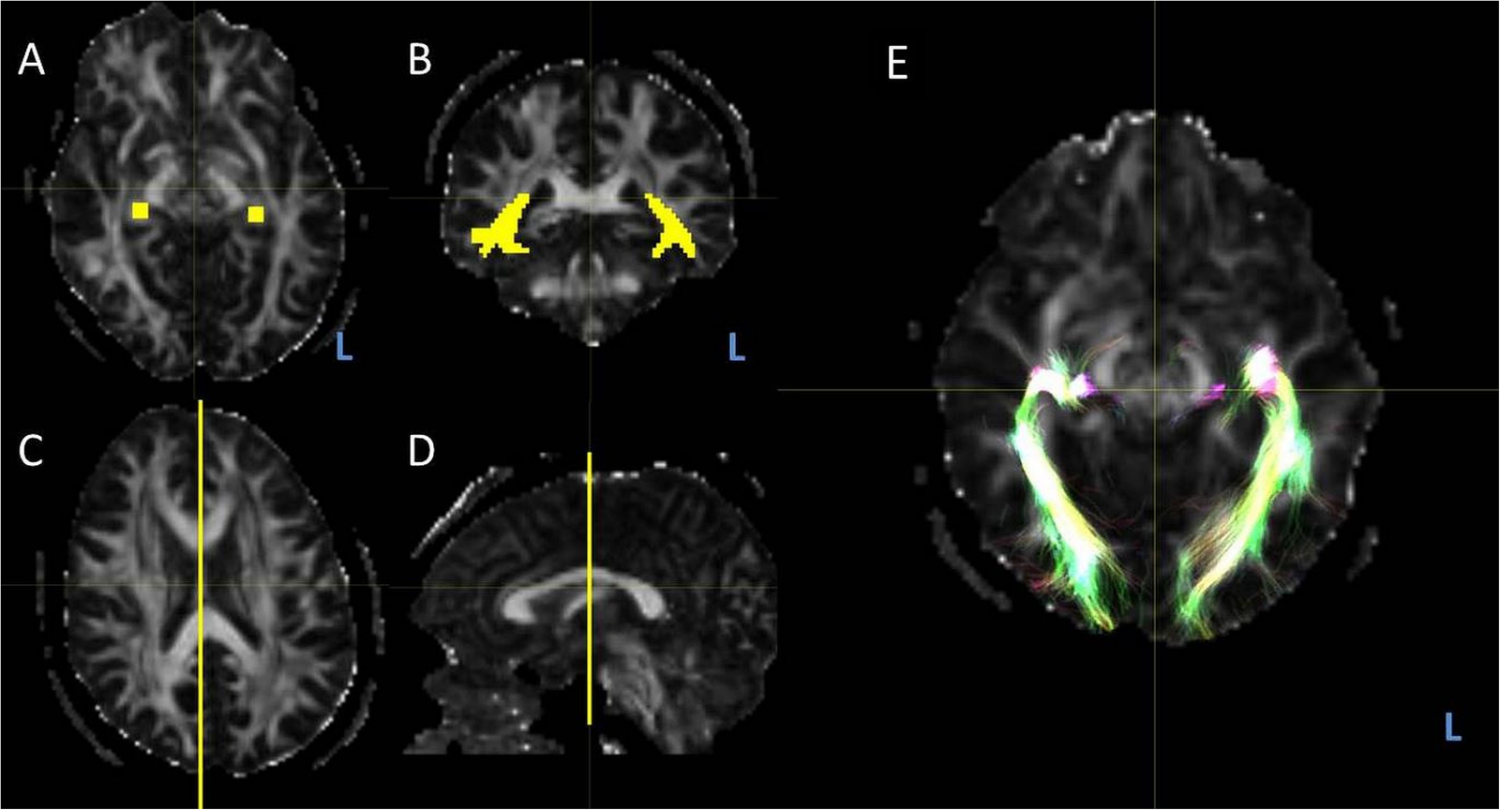
Region of interests, indicated in yellow, for optic radiation tractography. Regions of interest were generated in native space on the FA map in MRtrix. L, left. (A) The seed region was the lateral geniculate nucleus, identified on axial slices of the FA map by locating the optic chiasm and following the postchiasmal optic tracts posteriorly as they enter the thalamus. This was corroborated by identifying the transition of the posterior limb of the internal capsule to the cerebral peduncle. A generous seeding was undertaken, incorporating 4×4 voxels over 2 consecutive axial slices. (B) The waypoint was the stratum sagittale, identified posterior to the splenium of the corpus callosum at the level of the occipital horns of the lateral ventricle on the sagittal plane. Generous seeding was undertaken in a single coronal plane, with particular attention to include the inferior bundles that derive from the anterior Meyer's loop. (C) A midline exclusion mask in the sagittal plane was added to exclude apparent tracts that cross the midline. (D) Tractography was run with these regions of interest to determine the anterior extent of the Meyer's loop. A frontal exclusion mask in the coronal plane was then added to remove artefactual connexions to adjacent white matter tracts such as the inferior longitudinal fasciculus and the uncinate fasciculus. (E) Resulting tractography after addition of frontal exclusion mask.

**Figure 2:**
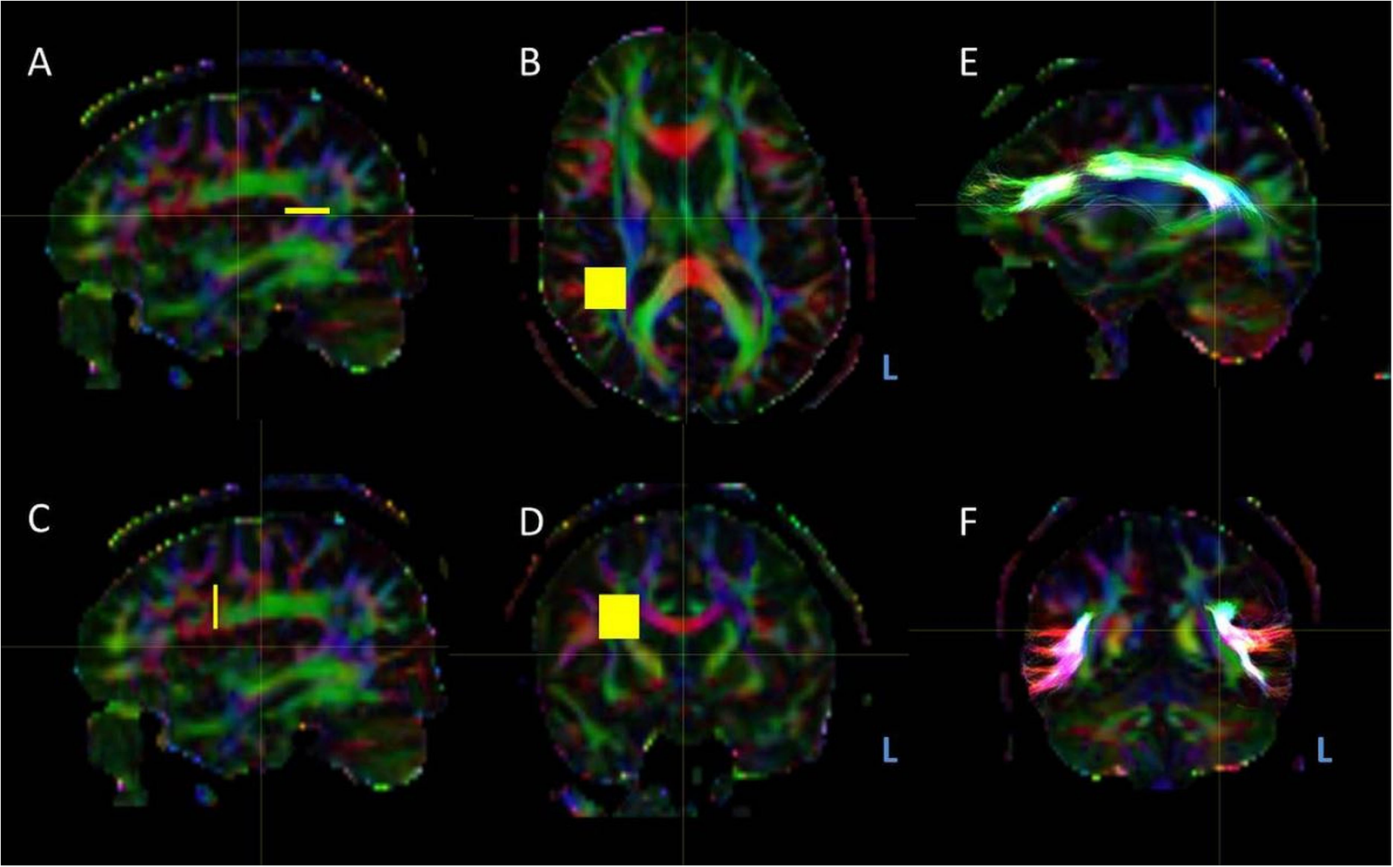
Regions of interest, indicated in yellow, for arcuate fasciculus tractography. Regions of interest were generated in native space on directionally-encoded colour map in MRtrix. L, left. (A and B) The seed region was the vertical limb of the fasciculus, identified as blue voxels, indicating vertical diffusion vectors, in the middle of the C-shaped tract on the sagittal planes of the coloured FA map (A). The seed is generated in the axial plane (B). (C and D) The waypoint was the inferior frontal gyrus, identified as red (left-right diffusion vectors) voxels in the sagittal plane of the coloured FA map (C). The seed is generated in the coronal plane (D). A midline exclusion mask was added to exclude connectivity that crossed the midline. (E and F) Resulting tractography of arcuate fasciculus in the sagittal and coronal plane (yellow).

Tractography was carried out using probabilistic tractography in MRTrix (using the SD_PROB command), using a minimum curvature radius of 1 mm, a step size of 0.2 mm, and a minimum Fibre orientation distribution (FOD) amplitude of 0.1, to generate 1000 tracts from the seed region that met all inclusion and exclusion criteria. Regions of interest used to carry out tractography of optic radiation and arcuate fasciculus are described in figures 1 and 2.

The resulting tract files were converted into a map of the fraction of tracks to intersect each voxel. The tract maps of arcuate fasciculi were thresholded at 5%, representing a compromise between retaining anatomically valid tracts and removing artefactual connexions. The optic radiations were exported in nifti format to EpiNav.(Centre for Medical Imaging and Computing, UCL, London). For display purposes, the optic radiations were thresholded individually, steadily increasing the threshold in an iterative process from 1% until the most anterior extent of the Meyer's loops begins to recede.

### Volumetric analyses

The volume of the arcuate fasciculus and optic radiation tracts were assessed in FSL using the thresholded tract maps. A measure of volumetric asymmetry of the arcuate fasciculus tracts was calculated using the formula:
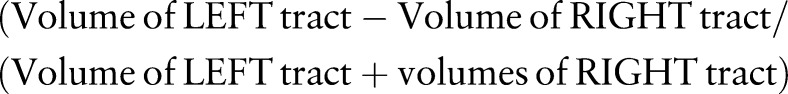


### Meyer's loop asymmetry

**Figure 3:**
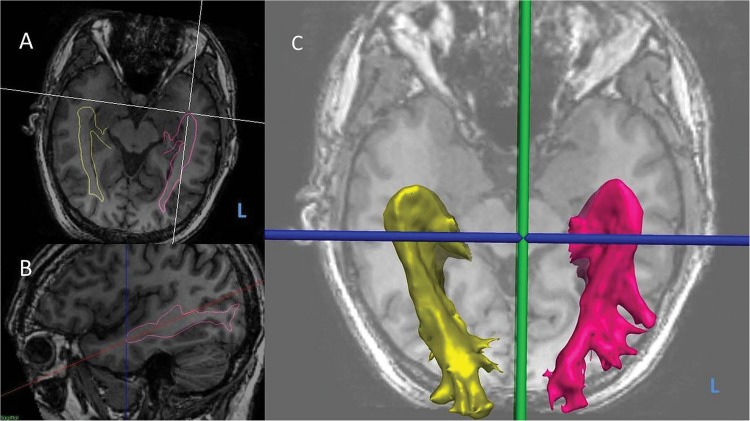
Measurement of optic radiation tractography asymmetry.L, left. (A) Oblique axial view of three-dimensional (3D) T1-weighted MRI with optic radiation tractography surface models (yellow-right, pink-left) overlaid. (B) Sagittal view of right optic radiation surface model (pink) with axial plane tilted to longitudinal axis of hippocampus, and Meyer's loop-temporal pole distance shown (dotted blue line). (C) 3D surface models of left and right optic radiations.

The distance from the anterior border of Meyer's loop to the temporal pole was measured in EpiNav. The FA map was rigidly coregistered to the 3D T1-weighted image, and used to warp the optic radiation tracts to the T1. The axial plane was tilted to run along the longitudinal axis of the hippocampi, and the distance from Meyer's loop to the temporal pole and from temporal horn to temporal pole was measured along this plane^10^ (figure 3).

Uncorrected Meyer's loop asymmetry was calculated using the formula:
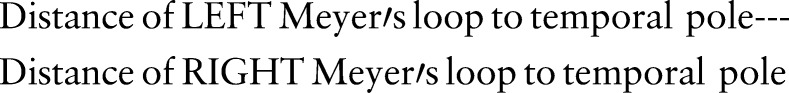


This value was corrected to account for interindividual variations in head size, using the formula:
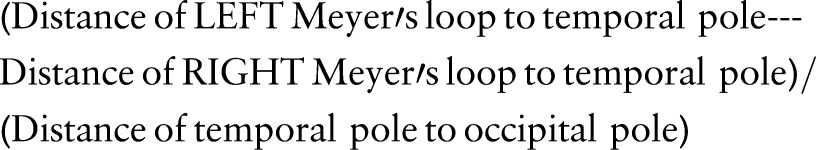


### Statistics

Patients were removed from analysis if there was a demonstrable failure of tractography to identify Meyer's loop due to the presence of large mass lesions. Remaining patients were grouped as left language dominant if LI was greater than 0.4 (left dominant), and non-left language dominant if LI was less than 0.4 (non-left dominant).^13^

Statistical analysis was performed with IBM SPSS software (V.22). Independent sample T tests were employed to look for significant differences between the groups with a Bonferroni correction for the two-prime hypotheses, and a Pearson correlation coefficient was calculated to assess for any linear correlations.

## Results

Intra-rater and inter-rater variability for tractography was assessed using mean DICE overlap of thresholded tracts from five subjects. The mean intra-rater DICE overlap was 0.76 for the arcuate fasciculus and 0.69 for the optic radiation. The mean inter-rater DICE overlap was 0.72 for the arcuate fasciculus and 0.74 for the optic radiation. These values are in the same range of, or higher than, scan-rescan reproducibility tests on CSD-based tractography.^25^ The intra-rater and inter-rater variability in the metrics derived from optic radiation tractography were assessed using intraclass correlation coefficients (ICC) between five subjects. The ICC for Meyer loop asymmetry and temporal horn asymmetry for the same user was 0.99 and 0.99 respectively, and between different users was 0.94 and 0.99.

**Figure 4:**
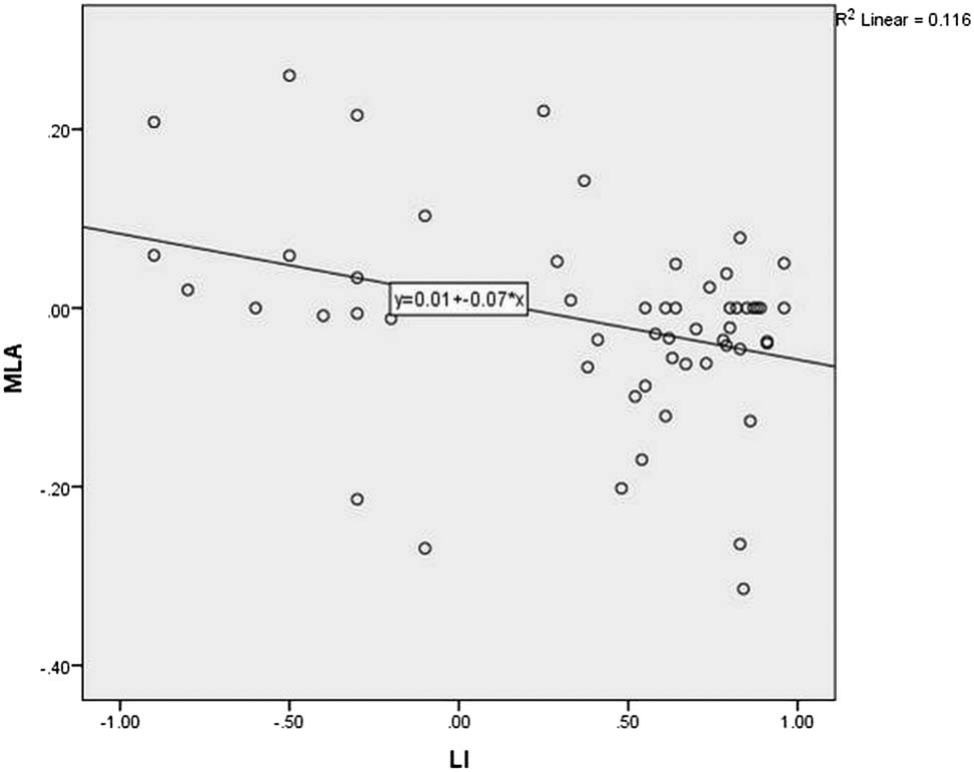
Scatterplot of corrected Meyer's loop asymmetry (MLA) against language lateralisation determined by functional MRI (LI). Verbal fluency LI +1=entirely left activation. −1=entirely right activation.

**Figure 5:**
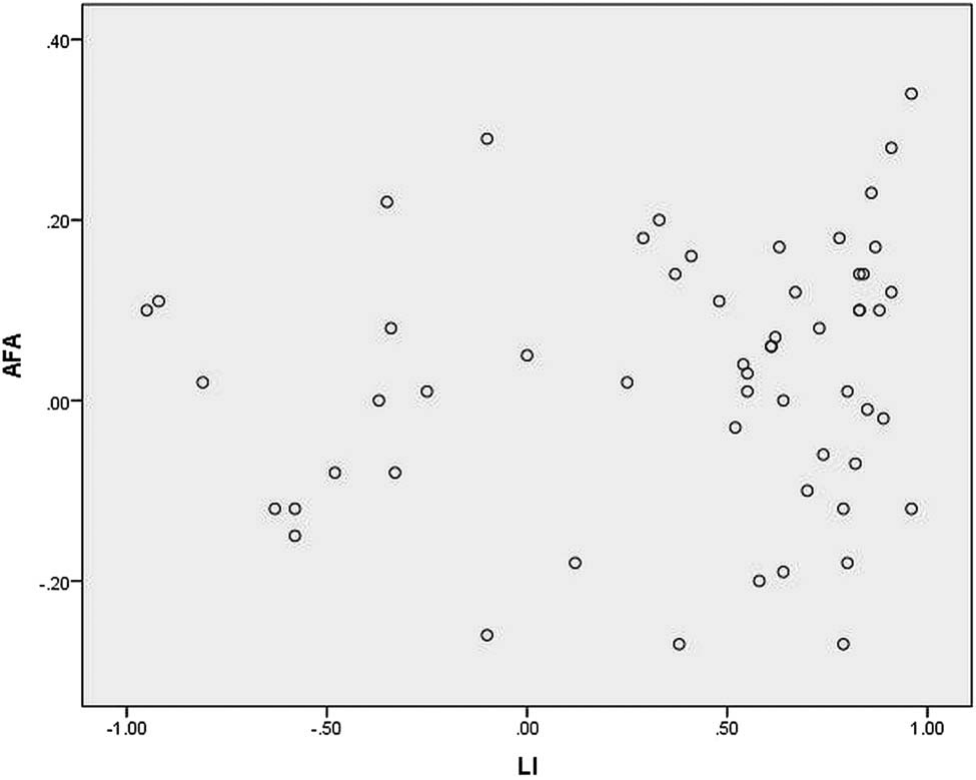
Scatterplot of arcuate fasciculus volume asymmetry (AFA) against language lateralisation determined by functional MRI (LI). Verbal fluency LI +1=entirely left activation. −1=entirely right activation.

Figure 4 shows the Pearson correlation for corrected MLA against language lateralisation in the whole group. There was a negative linear correlation, with greater left lateralised language associated with more anteriorly placed left Meyer's loops (R value −0.34, p=0.01) There was no linear correlation between language lateralisation and arcuate fasciculus asymmetry (figure 5) or temporal horn asymmetry.

In the total group, the mean distance from the anterior border of Meyer's loop to the temporal pole was 31.19 mm (SD 6.35 mm, range 19.5–48.8 mm) on the left and 32.10 mm (SD 6.34 mm, range 17.40–46.50 mm) on the right.

The mean distance of Meyer's loop behind the temporal horn was 3.28 mm (SD 7.18, range 20.10 mm behind to 28.80 mm in front) on the left, and 5.27 mm (SD 6.32, range 30.2 mm behind to 3.1 mm in front) on the right.

**Figure 6:**
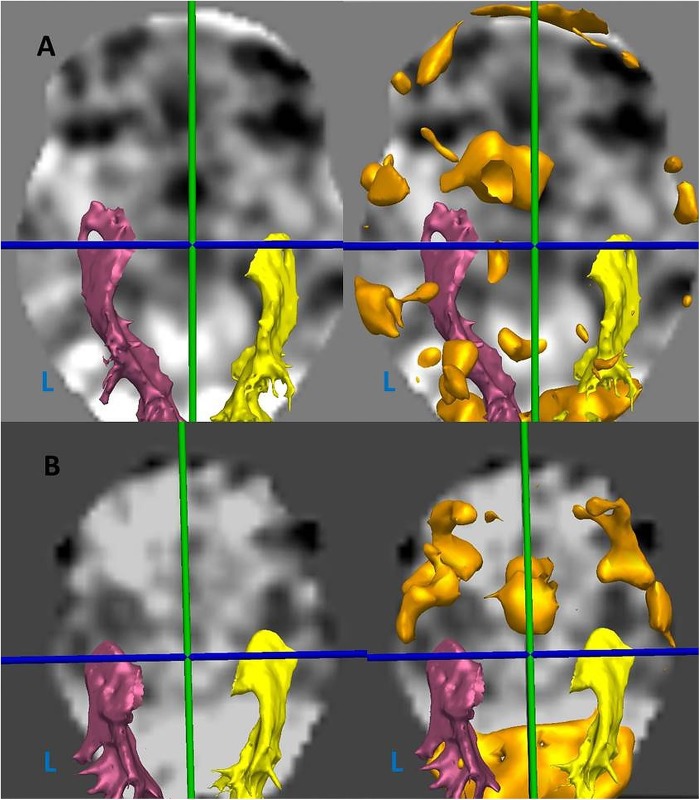
Axial view of fMRI statistical maps with optic radiation tractography (yellow- right, pink-left) and verbal fluency BOLD activation thresholded (orange) (L, left). (A) Patient with left-dominant language (LI 0.83), and anteriorly placed left Meyer's loop (MLA −14.9 mm). (B) Patient with non-left dominant language (LI 0.37), and posteriorly placed left Meyer's loop (MLA 0.7 mm).

The mean asymmetries in Meyer's loop, temporal horn and arcuate fasciculi volumes for the left and non-left language dominant groups are shown in table 2. There was a significant difference in corrected Meyer's loop asymmetry, with the left loop being anterior to the right loop in the LI>0.4 group, and posterior to the right loop in the LI<0.4 group (p=0.003; figure 6). There was no significant temporal horn asymmetry in either group. Arcuate fasciculus volumes were marginally greater on the left in both the LI>0.4 group (21 582 mm^3^ vs 19 934 mm^3^) and the LI<0.4 group (19 004 mm^3^ vs 17 633 mm^3^), with no significant difference between the LI>0.4 and LI<0.4 groups.

**Table 2:** Mean asymmetries in the left and non-left language groups

	LI>0.4		LI<0.4		
Variable	Mean	SD	Mean	SD	p Value
MLA (mm)	−2.78	7.47	2.64	8.92	0.02*
Corrected MLA	−0.46	0.083	0.40	0.12	0.003*
THA (mm)	0.71	11.54	1.89	8.75	0.69
AFA	0.04	0.14	0.01	0.16	0.42

*Statistical significance at p<0.025 with Bonferroni correction).

AFA, arcuate fasciculus asymmetry; Corrected MLA- corrected Meyer's loop asymmetry; LI, language lateralisation index; MLA-Meyer's loop asymmetry; THA- temporal horn asymmetry

No significant differences were seen in MLA when considering the lateralisation of the epilepsy (p=0.75), the presence of a lesion (p=0.71), or gender (0.93). An association between MLA and handedness is significant (p=0.024), reflecting the well-established relationship between handedness and language lateralisation.^26^ No correlation was seen with age (R 0.59, p=0.66) or duration of epilepsy (R −0.05, p=0.97).

## Discussion

### Summary of main findings

We performed optic radiation tractography in 57 subjects with TLE, using a probabilistic multifibre tractography method (constrained spherical deconvolution).

The mean distance of Meyer's loop to the temporal pole was 31 mm, although there was great variability between subjects, and also from one side to another.

In this study we examined the issue of MLA in a population with epilepsy and a reasonable distribution of language lateralisation determined by fMRI. We found a correlation between language lateralisation and MLA, with the left language dominant group having anteriorly placed Meyer's loops on the left side and the non-left language dominant group having anteriorly placed Meyer's loop on the right side. We found no correlation between arcuate fasciculus volume and language lateralisation.

### Comparison with previous data

There are a large number of previous optic radiation tractography studies, but these are limited by either low subject numbers or the use of deterministic tractography methods, which are recognised to underestimate the anterior extent of Meyer's loop.^21^ ^27^ ^28^ The group results in this study support the findings of dissection studies and other probabilistic tractography studies in terms of the anterior extent and variability of Meyer's loop in all subjects.^28^ ^29^

In the left language dominant group we found anteriorly placed Meyer's loops on the left side, with a mean distance of 30.3 mm from the temporal pole on the left and 33.1 mm on the right, giving an asymmetry of −2.8 mm. This is broadly consistent with the findings of a previous deterministic study, which showed a mean of 39.7 mm on the left and 45.5 mm on the right, and an asymmetry of −5.8 mm.^11^ However, the use of deterministic tractography in their study is likely to have resulted in an underestimation of the anterior extent of Meyer's loop. This is also consistent with the finding that visual field defects are more commonly seen with left-sided anterior temporal lobe resections, as the majority of patients are left language dominant.^12^

In the non-left language dominant group, we found that Meyer's loop is posteriorly placed on the left side in the non-left language group, with a mean distance of 32.8 mm on the left and 30.3 mm on the right, giving an asymmetry of 2.6 mm. We are not aware of previous reports of MLA in a non-left language dominant group.

In this study we did not find a linear relationship between the arcuate fasciculus tractography volumes and the language lateralisation indices. This is in agreement with previous earlier work,^7^ which suggests that functional lateralisation correlates better with the number of streamlines in the arcuate fasciculus and the fractional anisotropy. However, more recent studies have demonstrated a correlation between arcuate fasciculus volumes and language lateralisation in both healthy subjects^5^ ^30^ and patients.^4^ Interpretation of these studies is limited by the low number of subjects with atypical language lateralisation, which makes up a large proportion of our own series.

### Limitations

In this study we used verbal fluency fMRI to determine measures of language lateralisation. This is a well-established paradigm that gives reasonable correlation with the WADA test in patients with epilepsy.^31–33^ However, it is limited in that it represents frontal hemispheric specialisation with no reliable temporal activation. Recognising this we are developing auditory and visual naming paradigms that primarily activate temporal neocortex and this will lead to a future comparative study with the anatomy of temporal lobe structures. We anticipate finding a stronger association between temporal activation and MLA.

A further limitation to this study is that the subjects all had focal epilepsy, and do not represent the healthy population. It is established that epilepsy causes white matter changes, associated with the development of epileptogenic networks.^34^ For example, patients with temporal lobe epilepsy exhibit a global reduction in the volume of white matter tissue across frontal, temporal and parietal but not occipital lobe regions.^35^ It is possible that localised microstructural anatomofunctional disturbances present in epilepsy may affect both language lateralisation and MLA and may not be seen in the normal population. It is therefore necessary to evaluate this association in healthy subjects.

A possible confounding factor in our series is that our tractography of the arcuate fasciculus may have failed to differentiate between different components.^4^ The well-established direct pathway typically shows strong leftward lateralisation in right-handed subjects, whereas the indirect pathway, consisting of the anterior and posterior segments, show rightward and no lateralisation respectively.^3^ Although there is evidence that seizure networks affect arcuate fasciculi bilaterally^36^ a more tailored approach to tractography may give a different correlation with language lateralisation in patients with epilepsy.

### Neurobiological implications

The temporal horn is not a reliable marker for the anterior extent of Meyer's loop. Meyer's loop is commonly posterior to the temporal horn although there are subjects in whom it extends anterior to the temporal horn. Thus the surgeon has to take an individually tailored approach to anterior temporal lobe resections in order to avoid injury to the optic radiation, and a consequent visual field defect and this may be aided by the use of preoperatively or intraoperatively acquired tractography^29^

The finding that MLA may be associated with language lateralisation, as determined by verbal fluency fMRI, supports the hypothesis that language lateralisation is related to the architecture of the anterior temporal lobe. This adds to the literature on brain asymmetry, and the relationship between functional and structural lateralisation. We cannot ascribe cause and effect, or conclude whether language dominance influences the anatomy of the optic radiation in the anterior temporal lobe or vice-versa. However, there is growing evidence that lateralisation begins at the level of molecular genetic level,^37^ ^38^ and continues through to asymmetries in microcircuitry^39^ and processing.^40^ The observations of functional and macrostructural lateralisation are likely to reflect the end points of these complex developmental processes.

## Conclusions

In conclusion we present further evidence for intrasubject asymmetry in the anterior extent of Meyer's loop, and demonstrate a linear relationship between the extent of this asymmetry and the lateralisation of language determined by verbal fluency fMRI in patients with epilepsy. Further work should aim to evalaute this association in a healthy control population and to employ other language paradigms that primarily activate the temporal lobe.
